# How to estimate kinship

**DOI:** 10.1111/mec.14833

**Published:** 2018-09-07

**Authors:** Jérôme Goudet, Tomas Kay, Bruce S. Weir

**Affiliations:** ^1^ Department of Ecology and Evolution University of Lausanne Lausanne Switzerland; ^2^ Swiss Institute of Bioinformatics University of Lausanne Lausanne Switzerland; ^3^ Department of Biostatistics University of Washington Seattle Washington

**Keywords:** animal mating/breeding systems, behavior/social evolution, conservation genetics, quantitative genetics, wildlife management

## Abstract

The concept of kinship permeates many domains of fundamental and applied biology ranging from social evolution to conservation science to quantitative and human genetics. Until recently, pedigrees were the gold standard to infer kinship, but the advent of next‐generation sequencing and the availability of dense genetic markers in many species make it a good time to (re)evaluate the usefulness of genetic markers in this context. Using three published data sets where both pedigrees and markers are available, we evaluate two common and a new genetic estimator of kinship. We show discrepancies between pedigree values and marker estimates of kinship and explore via simulations the possible reasons for these. We find these discrepancies are attributable to two main sources: pedigree errors and heterogeneity in the origin of founders. We also show that our new marker‐based kinship estimator has very good statistical properties and behaviour and is particularly well suited for situations where the source population is of small size, as will often be the case in conservation biology, and where high levels of kinship are expected, as is typical in social evolution studies.

**Abbreviations**
Ibd: identity by descent
MAF: minor allele frequency
SNP: single nucleotide polymorphism

## 1 INTRODUCTION

Kinship, also known as coancestry or half‐relatedness, is important to many fields of biology (Csilléry et al., 2006; Speed & Balding, 2015). It is central to Hamilton's rule which explains how social behaviours evolve and how life went through major transitions in evolution (Fisher, Cornwallis, & West, 2013; Hamilton, 1963).

In conservation science, kinship between individuals is carefully documented for successful captive breeding programmes, and inbreeding (a function of self‐kinship) is measured to establish the extinction risk of threatened populations (Kleiman et al., 1986; Madsen, Stille, & Shine, 1996). Human geneticists control for kinship when conducting large‐scale GWASs to identify candidate genes involved in particular disorders (Campos, Gianola, & Allison, 2010; Harold et al., 2009; Hindorff et al., 2009; Rivas et al., 2011).

Following the advent of the genomic revolution, increasingly large genetic data sets have become available. Various ways these data can be used to estimate kinship have been proposed and are used currently (Li, Weeks, & Chakravarti, 1993; Lynch, 1988; Lynch & Ritland, 1999; Queller & Goodnight, 1989; Ritland, 1996; VanRaden, 2008; Wang, 2011; Yang et al., 2010). The most frequently used estimator varies from field to field: gcta (Genome‐wide Complex Trait Analysis) (Yang et al., 2010) and Ritland (1996) estimators are commonly used by human geneticists, while Queller‐Goodnight's (Queller & Goodnight, 1989) is common in conservation biology and social evolution (Weir & Goudet, 2017). The growing availability of large numbers of single nucleotide variant data has brought a need for reexamination of these standard estimators (Druet and Gautier, 2017; Griesser, Halvarsson, Drobniak, & Vilà, 2015; Kardos, Luikart, & Allendorf, 2015; Kardos, Taylor, Ellegren, Luikart, & Allendorf, 2016; Robinson, Santure, DeCauwer, Sheldon, & Slate, 2013a; Robinson, Simmons, & Kennington, 2013b; Santure et al., 2010). We (Weir & Goudet, 2017) recently offered a new set of estimators that were constructed to be relative to the population from which the study individuals were sampled. Here, we give a further examination of these new estimators, using simulated data and data from natural and domestic animal populations. On the basis of this examination, we recommend their use in several situations.

Identity by descent (ibd) is defined relative to a reference population, where different alleles are considered to be not ibd (Wang, 2016). Estimation rests on translating observed genotypes, reflecting allelic identity in state, into statements about allelic identity by descent. These translations generally require allele frequencies, meaning that both inbreeding and kinship estimation imply reference populations for allele frequencies. A common estimation approach (Milligan, 2003; Thompson, 1975) is to use allele frequencies in the current population as surrogates for reference population values, with the justification that “realistic samples will often involve enough individuals that errors in the allele‐frequency distribution will be quite small” (Milligan, 2003). However, this assumes no inbreeding and low mean relationship in the sample Ritland (1996), and in areas where it can be obtained, it is recommended to use the frequencies determined from the founders only (VanRaden, Olson, Wiggans, Cole, & Tooker, 2011). Hall, Mercer, Phillips, Shaw, and Anderson (2012) provided iterative EM‐algorithm estimates for both reference allele frequencies and inbreeding coefficients. Other authors (Vogl, Karhu, Moran, & Savolainen, 2002) adopt a Bayesian approach with a prior distribution for allele frequencies and inbreeding coefficients and derivation of a marginal posterior distribution for the inbreeding coefficients.

We recognize the many advantages of likelihood‐based (including Bayesian) methods, but simplicity and computational issues lead us to concentrate on the method of moments for estimation. A feature of our approach (Weir & Goudet, 2017) is that independence of alleles at a single locus in the reference population need not be assumed, although we have not considered dependencies among loci.

Our approach thus allows the target individuals to be inbred and is in line with Powell, Visscher, and Goddard (2010) in taking the current population as the reference. Doing so makes estimation of allele frequencies straightforward. The resulting estimates of inbreeding and kinship coefficients, now relative to the current population, are no longer of ibd probabilities but are of differences of ibd probabilities. These differences can be negative. An analogy at the population level for inbreeding would be changing the focus from the total inbreeding coefficient *F*_IT_ to the within‐population coefficient *F*_IS_. There is an analogous change from total kinship to within‐population kinship for both populations and individuals. It is this within‐population kinship that we can estimate. We make explicit that the kinship of a pair of individuals is compared to the average for all pairs of individuals in the sample, so the average estimated kinship is zero. Similarly, the inbreeding coefficient of an individual is the within‐individual ibd probability relative to the average between‐individual‐pair ibd probability and the resulting values can also be negative. This is consistent with Yu et al. (2006) who spoke of “adjusting the probability of identity by state between two individuals with the average probability of identity by state between random individuals” in order to address identity by descent. Other kinship estimation methods that do not use allele frequencies (e.g., KING‐robust; Manichaikul et al. 2010) are estimating ibd between individuals relative to that within individuals.

Frentiu et al. (2008) compared the use of pedigree‐ and marker‐based kinship values to estimate quantitative genetic variances and covariances for a set of traits in a wild bird population. They describe the advantage of marker‐based methods as avoiding the difficulties of reconstructing pedigrees of wild populations, but they claimed only mixed success with those methods. This may have reflected the small number of markers or the low level of variance in relatedness in their study population. Santure et al. (2010) concluded that the best estimates of relatedness are likelihood‐based, although they reached this conclusion based on a very small set of markers and a pedigree of few individuals. Their work was before we presented our estimator of kinship (Weir & Goudet, 2017) which we found has the smallest bias and standard deviation for data from a small pedigree of 135 individuals.

Pedigrees (records of which parents sired which offspring) have been important historically and foundational in many of the aforementioned fields; however, they have significant limitations. First, for many study populations pedigree data are unavailable and would be impossible to acquire. Second, pedigrees are susceptible to human error such as unnoticed extra‐pair copulations and misidentified paternity (Goossens et al., 1998). While pedigree reconstruction from genetic markers is possible (Ramstetter et al., 2017), it remains a daunting and difficult task for nonmodel organisms (Städele & Vigilant, 2016).

A third limitation is that pedigree relatedness is the expected value of kinship between two individuals. The randomness with which a diploid individual transmits alleles to offspring results in actual kinship and inbreeding coefficients varying around the pedigree‐based expected values (Wang, 2016). For example, half‐siblings have a kinship coefficient of 0.125. Half‐siblings either do or do not receive copies of the same allele from their common parent, so their actual kinship coefficients are one or zero at any locus and the average over the genome of these 1's and 0's will be close to 0.125. Wang (2016) proposes using “gene dropping” (assigning unique identifiers to each allele present in the founders and following their fate through the pedigree) along the pedigree to obtain the actual kinship values. He showed that when genome size is small, these can differ substantially from the relatedness calculated from a pedigree. For large genomes, estimates of kinship obtained by gene dropping or from pedigrees are almost identical.

The final, and most fundamental, limitation of pedigrees is the assumption of equally related “founding” individuals. In most populations, this assumption is highly inaccurate. At first, this problem seems surmountable by extending the pedigree further back. However, this can be done *ad infinitum*; all pairs of individuals (even across species) share a common ancestor somewhere along the tree of life (Speed & Balding, 2015). As pedigrees stretch further back, kinship values tend towards 1 and become useless (Speed & Balding, 2015). We therefore need to truncate pedigrees at some point and create artificial ‘founders’. Where we chose to truncate the pedigree is arbitrary, and since this decision dictates pedigree‐based kinship values, these values are also arbitrary. For these reasons, Speed and Balding (2015) argue that pedigree‐based kinship should not be the gold standard of kinship estimation.

SNP array data for many species are readily available, and as the cost of sequencing is plummeting, large‐scale genotyping of populations of several thousand individuals will soon be possible, as is already the case for human populations and a few other domesticated species (e.g., cattle). The number of single nucleotide variants required to provide satisfactory estimates of the kinships among these individuals remains unknown. The aim of this study was threefold: (a) to examine the properties of kinship estimators for real pedigrees of varying size, structure and completeness; (b) to explore, using simulations, the causes underlying different estimator properties; and (c) to evaluate the usefulness of pedigree‐based estimates of kinship.

## 2 METHODS

### 2.1 Allele‐pair matching estimates

#### 2.1.1 Pedigree values

For individual *j*, the inbreeding coefficient *F*_*j*_ is the probability that its two alleles at a given locus are ibd. The kinship, or coancestry, coefficient *θ*_*jj*′_ for individuals *j* and *j*′ is defined here as the average of the four ibd probabilities for one allele from each individual. It follows that the kinship of individual *j* with itself is (1 + *F*_*j*_)/2. Generally, however, we reserve the term kinship for distinct individuals. *θ*_S_ denotes the average of the kinships over pairs of individuals for (samples from) a population.

If individual *J* is ancestral to individuals *j* and *j*′, and if there are *n* individuals in the pedigree path joining *j* to *j*′ through *J*, including *j* and *j*′, then *θ*_*jj*′_ = ∑(0.5)^*n*^(1 + *F*_*J*_), where *F*_*J*_ is the pedigree inbreeding coefficient of *J* and the sum is over all ancestors *J* and all paths joining *j* to *j*′ through *J* (Wright, 1922). The pedigree kinship *θ*_*jj*′_ is also the inbreeding coefficient of an individual with parents *j* and *j*′. If ancestor *J* is further back in time than the time of the reference population, then it is assumed that it does not contribute to the relatedness of individuals *j* and *j*′. These kinships are predicted values from pedigrees. The (often unstated) reference for predicted kinship values from pedigrees is the set of founders, who are assumed to have a kinship of 0 with other founders.

#### 2.1.2 Marker‐based estimates

We (Weir & Goudet, 2017) adopted a method‐of‐moments estimate for the kinship coefficient *θ*_*jj*′_ for individuals *j* and *j*′ in a sample of individuals *relative to* the average kinship *θ*_S_ of all pairs of individuals in the sample. Making estimates “relative to” meant that the reference allele frequencies did not need to be estimated. It also meant that kinship estimates for pairs of individuals who share less alleles than the population average are negative. Instead of estimating the total kinship coefficient *θ*_*jj*′_, we focus on the within‐population parameter β_*jj*′_ = (*θ*_*jj*′_ − *θ*_S_)/(1 − *θ*_S_). This comes from the following relationship for individual‐level kinship coefficients: (1 − *θ*_*jj*′_) = (1 − β_*jj*′_)(1 − *θ*_S_), analogous to the well‐known relation for population‐level inbreeding coefficients (Wright, 1922): (1 − *F*_IT_) = (1 − *F*_IS_)(1 − *F*_ST_). This leads to a discrepancy between marker‐based estimates (which can be negative) and pedigree‐based expectations (which range from 0 to 1).

In Weir and Goudet (2017), we wrote the new estimator as $\hat{\beta_{jj^{\prime }}}$, but now we write $r_{jj^{\prime }}^{\beta }$ for consistency with the ecological literature (Wang, 2016). $r_{jj^{\prime }}^{\beta }$ can be calculated with the r package hierfstat (Goudet, 2005) and is defined as follows:

(1) $$r_{jj^{\prime }}^{\beta }=\frac{M_{jj^{\prime }}-M_{S}}{1-M_{S}}$$

where *M*_*jj*′_ is the proportion of alleles carried by individuals *j* and *j*′ that are identical in state, that is, match. *M*_*jj*′_ can be conveniently computed as $\left\{\sum_{l=1}^{L}\left[1+\left(X_{j_{l}}-1\right)\left(X_{j_{l}^{\prime }}-1\right)\right]/2\right\}$, where $X_{j_{l}}$ is the dosage of a particular allele, for example, reference allele, at marker *l* for individual *j*. For a sample of *n* individuals, *M*_S_ is the average matching for all pairs of distinct individuals:

$$M_{S}=\frac{1}{n(n-1)}\underset{j\neq j^{\prime }}{\sum_{j=1}^{n}\sum_{j^{\prime }=1}^{n}M_{jj^{\prime }}}$$

It is difficult to derive the sampling distribution of ratios of second‐order statistics, but we note that the ratio of the expected values of the numerator and denominator of $r_{jj^{\prime }}^{\beta }$ is the target parameter (*θ*_*jj*′_ − *θ*_S_)/(1 − *θ*_S_), and we showed Weir and Goudet (2017) that the estimator has low mean square error for the target parameter. We have also found (data not shown) that the estimator behaves better with larger numbers of SNPs.

For a single individual, *j* = *j*′, our estimate is for [(1 + *F*_*j*_)/2 − *θ*_S_]/(1 − *θ*_S_), and an estimator of *F*_*j*_ relative to the average kinship is $\left(2r_{j}^{\beta }-1\right)$. We note that *M*_*jj*′_ is the central quantity in the third estimator of VanRaden (2008), and thus, this third estimator and $r_{jj^{\prime }}^{\beta }$ are colinear.

2.1.3

Other moment estimators, such as that of Ritland (1996), in effect assume sample allele frequencies ($\tilde{p}$) for the reference allele at a locus can be used in place of the reference population allele frequencies. Details for two such estimators that differ in how they combine information over loci now follow (Speed and Balding 2015 suggest other ways of combining information across loci, but all these weightings assume sample allele frequencies can be used in place of the reference population allele frequencies).

When information is combined over loci by weighting with sample heterozygosities, we write a common kinship estimator as $r_{jj^{\prime }}^{w}$:

(2) $$r_{jj^{\prime }}^{w}=\frac{\sum_{l=1}^{L}\left(X_{j_{l}}-2{\tilde{p}}_{l}\right)\left(X_{j_{l}^{\prime }}-2{\tilde{p}}_{l}\right)}{2\sum_{l=1}^{L}{\tilde{p}}_{l}\left(1-{\tilde{p}}_{l}\right)}$$

The weighted estimator in Equation (2) is the first estimator discussed by VanRaden (2008). It estimates (1 + *F*_*j*_)/2 when *j* = *j*′ and *θ*_*jj*′_ when *j* ≠ *j*′. There is no simple translation from these estimates to those we propose in Equation (1).

It is common to refer to $\left(X_{j_{l}}-2{\tilde{p}}_{l}\right)/\sqrt{\left[2{\tilde{p}}_{l}\left(1-{\tilde{p}}_{l}\right)\right]}$ as a standardized genotype measure on the basis that the expected value of $X_{j_{l}}$ is twice the allele frequency ($2p_{l}$) in the reference population. However, the variance of $X_{j_{l}}$ is $2p_{l}\left(1-p_{l}\right)(1+F_{j}$) rather than $2p_{l}\left(1-p_{l}\right)$.

We have focused on the kinship for individuals *j*,* j*′ relative to that for all pairs of individuals in a sample. Another perspective is implicit in the use of the estimators $r_{jj^{\prime }}^{w}$ and $r_{jj^{\prime }}^{u}$. There the kinship for the target pair of individuals measures the additional probability of ibd of each individual with every other sample member over that between all sample pairs. This takes account of the different pedigrees of each of the target pairs: If ψ_*j*_ is the average kinship of *j* with every other individual in the sample, ψ_*j*_ = ∑ _*j*″≠*j*_ *θ*_*jj*″_/(*n* − 1), then the target parameter with this perspective is:

(3) $$\gamma_{jj^{\prime }}=\frac{\theta_{jj^{\prime }}-\psi_{j}-\psi_{j^{\prime }}+\theta_{S}}{1-\theta_{S}}$$

We showed in simulations (Weir & Goudet, 2017) that, in fact, $r_{jj^{\prime }}^{w}$ is a good estimator for this new parameter and $r_{jj^{\prime }}^{u}$ less so.

For large sample sizes, we note that the ratio of the expected values of the numerator and denominator of $r_{jj^{\prime }}^{w}$ is indeed the new parameter. It is not clear when γ_*jj*′_ (Equation (3)) will be preferable to β_*jj*′_ = (*θ*_*jj*′_ − *θ*_S_)/(1 − *θ*_S_), but clearly neither is the same as the pedigree‐based value of *θ*_*jj*′_.

When information over loci is combined as an unweighted average, we write a common kinship estimator as $r_{jj^{\prime }}^{u}$:

(4) $$r_{jj^{\prime }}^{u}=\frac{1}{L}\;\sum_{l=1}^{L}\frac{\left(X_{j_{l}}-2{\tilde{p}}_{l}\right)\left(X_{j_{l}^{\prime }}-2{\tilde{p}}_{l}\right)}{2{\tilde{p}}_{l}\left(1-{\tilde{p}}_{l}\right)}$$

These terms correspond to the second estimator of VanRaden (2008), and they form the off‐diagonal elements of the genetic relatedness matrix in GCTA (Yang, Lee, Goddard, & Visscher, 2011). We note that VanRaden (2008) called this estimator “weighted,” because in his matrix notation, the diagonal matrix D of locus variances comes between the dosage matrices X and $X^{\prime }$ (M and $M^{\prime }$ in the notation of VanRaden 2008, respectively).

### 2.2 Real data sets

We illustrate the properties of the three estimators *r* ^β^, *r* ^w^ and *r* ^u^ with published data sets from great tits (Robinson et al., 2013a), sheep (Bérénos, Ellis, Pilkington, & Pemberton, 2014) and domestic pigs (Cleveland, Hickey, & Forni, 2012) for which both pedigree and genetic data are available.

#### 2.2.1 Great tit data set

Published in Robinson et al. (2013a), the data consist of a pedigree of 2,497 individuals and their genotypes at 5,591 SNPs. Of the 2,947 individuals, 1,177 are founders. Of the remaining 1,240 individuals, 53 have unknown sires and two have unknown dams. The number of SNPs is fairly limited, and the pedigree is quite shallow; the longest loop is five ancestors (three generations) long.

#### 2.2.2 Soay sheep data set

Described in Bérénos et al. (2014), the data consist of two pedigrees obtained by different means, and the genotypes of about half the pedigreed animals. We use only the second and more complete pedigree. It was built using SNP assignment for both paternity and maternity whenever possible and complemented with microsatellite‐assigned parentage; otherwise, see Morrissey et al. (2012). The pedigree consists of 6,740 individuals, of which 404 are founders. Of the remaining individuals, 355 have missing dams and 1,743 have missing sires, making 33% missing individuals. A total of 3,973 individuals have been genotyped at 34,538 SNPs. The distribution of the number of offspring for dams and sires is noteworthy. Numbers range from 1 to 20 lambs per dam and from 1 to 107 lambs per sire, with a very skewed distribution. This pedigree is quite deep; the longest loop is 18 ancestors (nine generations) long.

#### 2.2.3 Pig data set

Described in Cleveland et al. (2012), the data consist of a pedigree of 6,473 individuals with 1,247 founders and 1,011 sire and 3,102 dam parents. Of these 6,473 individuals, 55% (3,534 pigs, including 81 founders) have been genotyped at 52,843 SNPs. The mean number of piglets per sire is 5.17 (SD = 7.06) and per dam is 1.68 (SD = 1.19). What makes this data set particularly useful is its completeness: Apart from the founders, all individuals have known parents. The longest loop in the pedigree is 17 ancestors long, and thus, the pedigree spans at least eight generations.

#### 2.2.4 Real data analyses

**Figure 1:**
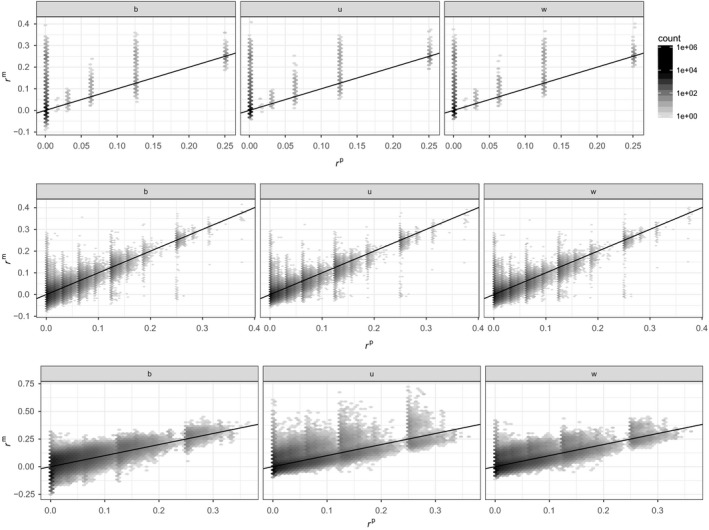
Density plot (hexagons) of marker‐based kinship estimates as a function of pedigree‐based predictions (the darker the hexagon, the more points, and density is on a log 10 scale). Top row: great tit data; middle row: Soay sheep data; and bottom row: pig data. Left column: *r* ^β^; middle column: *r* ^u^; and right column: *r* ^w^. For all panels, the black line is the one‐to‐one relationship

Figure 1 shows the relation between the three marker‐based estimators and pedigree kinship. The top row corresponds to the tit data set, the middle row to the sheep data set and the bottom row to the pig data set.

Across all three data sets, and for all three marker‐based estimators, the correlation between the three marker‐ and pedigree‐based kinship is noisy, the more so the smaller the proportion of individuals genotyped (from bottom to top). Assuming there is some ‘correct’ value of kinship, this noise could be due to inaccuracies in the pedigree‐based expectations, in the marker‐based estimates, or in both. Note that in the tit and sheep data sets, some genotypes are missing, while they have been imputed in the pig data set. We described in supplementary materials how to handle missing data when estimating *r* ^β^. For *r* ^w^ and *r* ^u^, we used the solution implemented in gcta (Yang et al., 2010).

For the sheep and pig data sets, we have also simulated genetic data along the observed pedigrees and we contrast the results obtained from real genotypes to those obtained from simulated ones. Genotypes were simulated at 88*k* SNPs for founders and unknown parents using the program ms (Hudson, 2002), and assuming they all came from a large random‐mating population. More details on the simulations can be found in the supplementary material. The alleles at each of these loci were then dropped along the observed pedigree (assuming each locus was independent) to obtain simulated genotypes for individuals with known parents.

### 2.3 Simulated pedigrees

To understand the results in Figure 1, we used simulations to explain the behaviour of the three estimators. We generated pedigrees according to two mating systems: random mating and monogamy. The pedigrees extended over five generations, and we varied the number of founders. The number of offspring for each generation was drawn from a Poisson distribution, so the exact number of individuals in a pedigree will vary, but is expected to be six times the number of founders. Genotypes for 2,000 founders at 88*k* SNPs were generated using ms, assuming a single random‐mating population at equilibrium between mutation and drift. ms generates haplotypes, and we combined two randomly chosen haplotypes to create diploid genotypes.

#### 2.3.1 Mating systems

Under random mating, the total number of offspring for a given generation was obtained by drawing *n*_f_/2 times (where *n*_f_ is the number of founders) from a Poisson distribution with parameter λ = 2. For each offspring, the two parents were drawn at random and without replacement. Contrastingly, under monogamy, two parents were drawn at random and had their number of offspring drawn from a Poisson distribution with λ = 2. Thus, more individuals will be full‐sibs in a monogamous population than in a random‐mating population.

#### 2.3.2 Number of founders

For each mating system, we simulated pedigrees with between 20 and 1,000 founders. We varied founder number incrementally: in steps of 10 between 20 and 100, in steps of 50 between 100 and 250 and in steps of 250 between 250 and 1,000. For each mating system and number of founders, we simulated 10 pedigrees.

### 2.4 Simulated data analyses

For each of the pedigrees and associated genetic data sets, we report the correlation between the kinships estimated with markers (*r* ^β^, *r* ^w^ and *r* ^u^) and those expected from pedigrees (*r* ^p^). We show the correlations as a function of the standard deviation in pedigree kinship SD(*r*_p_) among all individuals in the pedigree: If there were no variation in pedigree kinship, as would be the case in an infinite random‐mating population, pedigree‐based kinship values should not correlate with marker‐based estimates. As the standard deviation in pedigree kinship increases (when the population gets smaller and/or the mating system creates more related individuals), correlation between marker‐based estimates and pedigree values should increase. We therefore expect an increase in the correlation between pedigree values and marker‐based estimates as the standard deviation in pedigree kinship increases (for smaller populations and populations with mating systems that produce more relatives). We show in Supporting Information Figure S1 that SD(*r*_p_) is a decreasing function of the number of individuals in the pedigree.

#### 2.4.1 Number of markers

A total of 88*k* loci were polymorphic among the 2*k* potential founders, but only 42*k* were polymorphic among the 20 founders of the smaller pedigree. With more markers, the precision of the marker‐based kinship estimates should increase. We investigated this effect by taking subsamples of 100, 500, 1*k*, 5*k*, 10*k*, 20*k* and 40*k* markers. To compare the effect of founder number, we used 42*k* markers.

#### 2.4.2 Origin of the founders

When calculating the pedigree kinship *r* ^p^, all founders are assumed to be unrelated. Marker‐based kinship estimate *r* ^β^ makes no such assumption, while *r* ^u^ and *r* ^w^ assume the genotyped individuals come from a random‐mating population. To investigate the effect of related founders, we simulated founders coming in equal proportion from two source populations (rather than from a single random‐mating population as above) using ms. These two populations were at equilibrium between mutation, migration and drift and exchanged one migrant per generation, and thus, *F*_ST_ between these two populations is 0.2. There is a large discrepancy between *r* ^p^ and *r* ^β^, *r* ^w^ and *r* ^u^ in this situation (see below).

A possible solution to this problem, if founder genotypes are available}, is to plug in the marker‐based kinship estimates of the founders, rather than assuming they are unrelated (see, for instance, Legarra, Aguilar, and Misztal (2009) and Misztal, Legarra, and Aguilar (2009)). We investigated whether doing so reduced the discrepancy between pedigree‐based predictions and marker‐based estimates of kinship.

## 3 RESULTS

### 3.1 Behaviour of marker‐based estimators in simulated pedigrees

**Figure 2:**
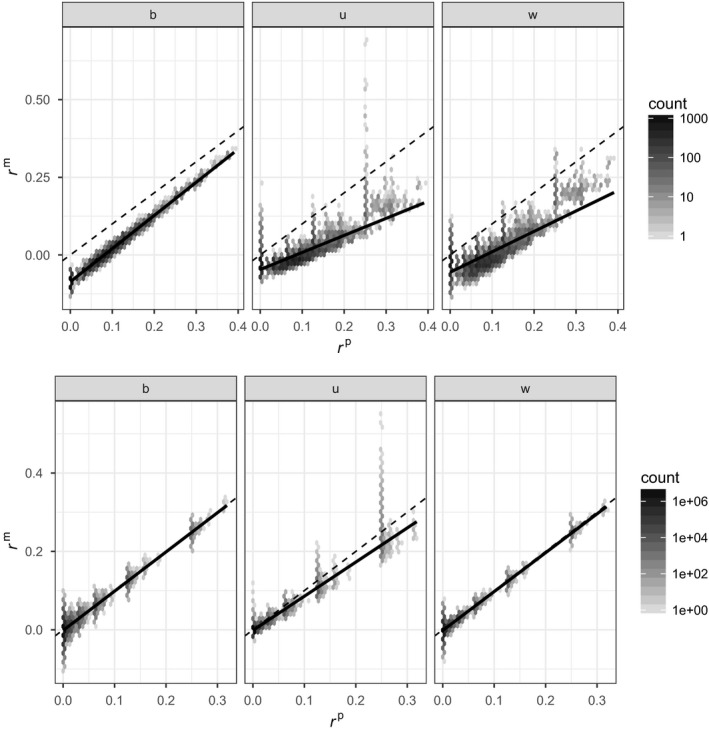
Density plot (hexagons) of marker‐based kinship estimates as a function of pedigree‐based expectations (darker means more points in the hexagon, and density is on log 10 scale). Top row: monogamous mating, 20 founders; and bottom row: random mating, 750 founders. Left column (b): *r* ^β^; middle column (u): *r* ^u^; and right column (w): *r* ^w^. Dotted line is the one‐to‐one relationship. Solid line is the regression between marker kinship and pedigree kinship

Figure 2 illustrates the relationship between marker‐based estimates and pedigree‐based kinship predictions for simulated data. The top row shows results for one of the 10 pedigrees generated with 20 founders and monogamous mating. The bottom row shows results for one of the 10 pedigrees generated with 750 founders and random mating. The density of points is represented as hexagons of varying darkness: the darker, the more numerous.

A cursory inspection of Figure 2 shows that all marker‐based estimates tend to underestimate the pedigree kinships when the number of founders is small (top row of Figure 2), and the three marker‐based estimates have different properties.

*r* ^β^ (left column of Figure 2) is an estimate of β and closely follows the pedigree values *r* ^p^ = *θ*, with a constant downward discrepancy for the pedigree with 20 founders (top left panel) and almost perfectly for the pedigree with 750 founders (bottom left). The downward discrepancy for *r* ^β^ is due to the constraint that the average of all *r* ^β^ is 0; this can easily be corrected (by imposing the same constraint on *r*_p_ and replacing it with $\left(r_{p}-\overset{¯}{r_{p}}\right)/\left(1-\overset{¯}{r_{p}}\right)$, where $\overset{¯}{r_{p}}$ is the mean kinship of all individuals in the pedigree), as shown in Weir and Goudet (2017). There is some scatter around the most common pedigree values of kinship (left column) corresponding to unrelated, half‐ and full‐sibs, and this more pronounced in the larger pedigree (bottom left panel).

*r* ^u^ estimates γ and underestimates the pedigree kinship *θ* (middle column of Figure 2), and this is more pronounced for large values of pedigree kinship, and for pedigrees with few founders (top) than those with many (bottom). For the full‐sib category (*r* ^p^ = 0.25), *r* ^u^ shows extreme scatter, and a similar effect, although less pronounced, is seen for half‐sibs (*r* ^p^ = 0.125). For the large pedigree, the relation between *r* ^u^ and *r* ^p^ for low kinship values is very tight (bottom middle panel), more so than for *r* ^β^.

The relation between *r* ^w^, another estimate of γ, and *r* ^p^ (right column of Figure 2) is noisy for the small pedigree (top right), with common pedigree kinship classes very spread out. *r* ^w^ also tends to underestimate *r* ^p^, the more so the larger the pedigree kinship. For the large pedigree (bottom right), the relation is much tighter; the regression slope is close to 1 and the scatter is less than for *r* ^β^. The extreme scatter for *r* ^p^ = 0.25 seen in *r* ^u^ for both the small and large pedigrees (middle column) is also seen in *r* ^w^ for small pedigrees (top right) but disappears when the pedigree is large (bottom right).

**Figure 3:**
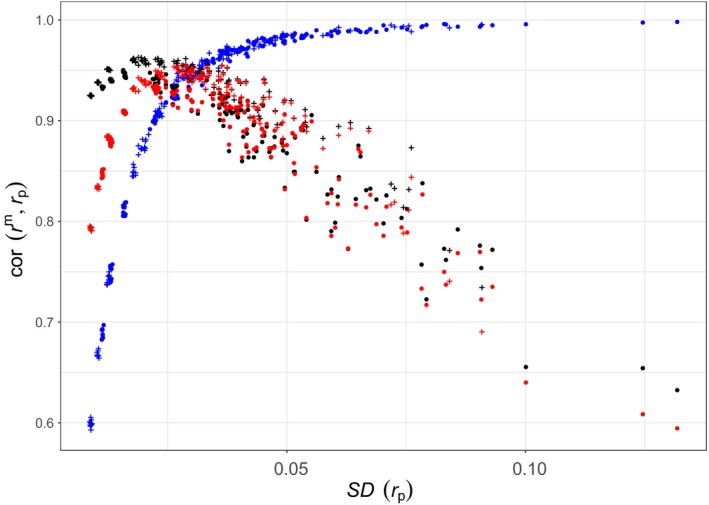
Correlation between marker‐based kinship with 42*k* SNPs and pedigree‐based prediction, against the standard deviation of pedigree‐based kinship. Each point corresponds to one of the 300 simulated pedigrees. Blue: *r* ^β^; red: *r* ^w^; and black: *r* ^u^. Filled circles: monogamous pedigrees. +: random‐mating pedigrees [Colour figure can be viewed at http://wileyonlinelibrary.com]

Figure 3 shows the correlations of *r* ^β^, *r* ^w^ and *r* ^u^ with *r* ^p^ (in blue, red and black, respectively) as a function of variation in kinship. Pairs of individuals from pedigrees with few founders will have more chance of being related than pairs from larger pedigrees. The pedigrees with few founders will thus have more variation in kinship and will be located to the right‐hand side of the graph, while pedigrees with many founders will be to the left. For a given number of founders, monogamous pedigrees (filled circles) will show more variation in kinship than random‐mating pedigrees (+ symbols) and will thus be located to their right.

The correlation between *r* ^β^ and *r* ^p^ shows a very different pattern from that of the other two marker‐based estimates: It increases as the variance in *r* ^p^ increases, while the correlations of *r* ^w^ and *r* ^u^ decrease as the standard deviation in *r* ^p^ increases above ≈ 0.02. The relation for the correlation between marker‐ and pedigree‐based kinship is very tight for *r* ^β^, with all the points falling on the same trajectory. This correlation is around 0.6 when the standard deviation in pedigree kinship is ≈ 0.009, and asymptotes at one as the standard deviation of pedigree kinship increases.

The pattern for *r* ^w^ and *r* ^u^ is almost the reverse. Correlation increases at first, when there is very little variance in pedigree kinship; reaches a maximum at around 0.93 for *r* ^w^ and 0.96 for *r* ^u^, when the standard deviation in pedigree kinship is between 0.02 and 0.03; and decreases linearly thereafter, although with greater scatter, as the standard deviation in *r* ^p^ increases above 0.03.

From this, it would seem that when the standard deviation in pedigree kinship is larger than ≈ 0.02 − 0.04, *r* ^β^ is a better estimator of pedigree kinship, while when the standard deviation in pedigree kinship is less than 0.03, *r* ^u^ is preferable (*r* ^u^ outperforms *r* ^w^ over the whole range). However, this is partly misleading as we saw in Figure 2 that *r* ^β^ is actually very close to *r* ^p^ over its whole range; *r* ^u^, while giving precise estimation for low values of *r* ^p^, is not very good in estimating the kinships of full‐ and half‐sibs.

The pattern observed for the correlation between marker and pedigree kinship for *r* ^w^ and *r* ^u^ is puzzling. The pattern observed with *r* ^β^ makes intuitive sense: If there is no variation in pedigree kinship, then it cannot be correlated with anything (correlation with a constant is 0 by definition). The larger the variation in pedigree kinship, the more there is to explain; thus, marker‐based estimates of kinship should be more correlated with pedigree values. We will return to this point later.

### 3.2 Number of loci

**Figure 4:**
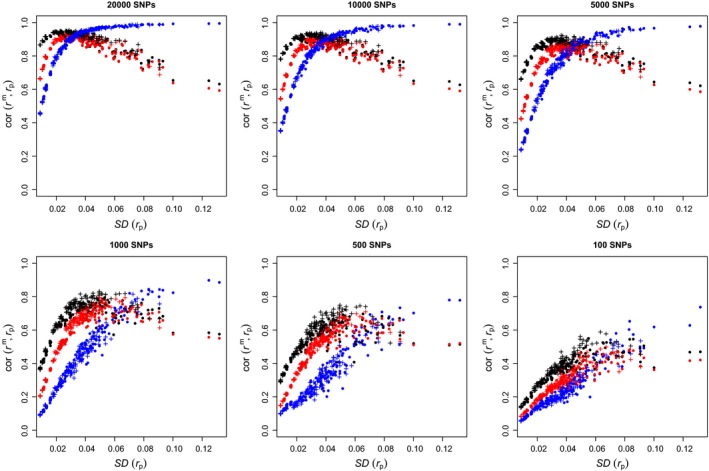
Correlations between marker‐ and pedigree‐based kinship, against the standard deviation of pedigree‐based kinship with different numbers of SNPs. Each point corresponds to one of 300 simulated pedigrees. Symbols and colours as in Figure3 [Colour figure can be viewed at http://wileyonlinelibrary.com]

In Figure 3, we fixed the number of (variable) SNPs to 42*k*. This is a fairly large number, though by no means exceptional nowadays. In Figure 4, we look at the effect of the number of markers, decreasing it to 20*k*, 10*k*, 5*k*, 1*k*, 500 and finally 100.

As the number of markers decreases, we see both quantitative and qualitative changes. The top row of Figure 4 (20*k*, 10*k* and 5*k* SNPs) shows slightly noisier versions of Figure 3, but otherwise no qualitative differences: For high variation in pedigree kinship, *r* ^β^ has a higher correlation with *r* ^p^ than *r* ^w^ or *r* ^u^, while for low variation, *r* ^w^ and *r* ^u^ are more correlated with *r* ^p^. Note that *r* ^w^ in particular reaches a plateau and starts decreasing when the variation in *r* ^p^ gets very low (for 10*k* and 5*k* SNPs).

The bottom row of Figure 4 (1*k*, 500 and 100 SNPs) looks different. First, the correlations between marker‐based estimates and pedigree‐based predictions are much lower, below 0.8 for 1*k*, 0.6 for 500 and 0.4 for 100 SNPs. Second, for the three marker‐based kinship estimators, correlation with pedigree‐based kinship increases as variation in pedigree kinship increases. In all three panels of the bottom row, the correlation between *r* ^β^ and *r* ^p^ is less than that for *r* ^w^, which itself is less than that for *r* ^u^.

### 3.3 Founders from two populations

**Figure 5:**
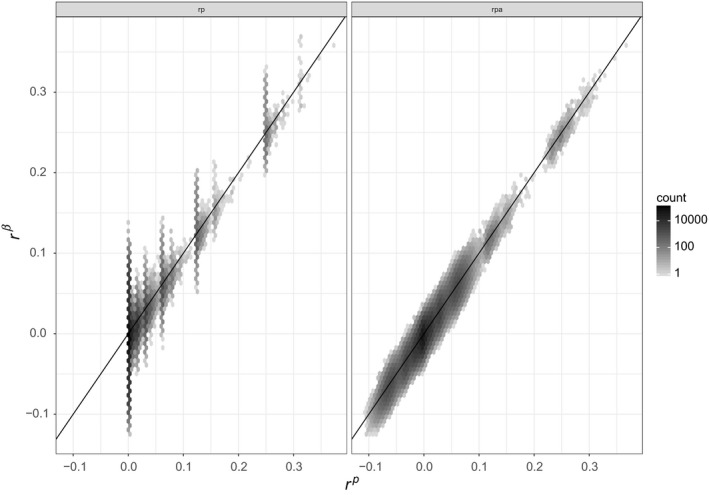
Marker kinship as a function of pedigree kinship when the founders come from two populations. Left panel: Founders are assumed unrelated (*r* ^p^). Right panel: Founders kinships have been estimated from markers ($r_{a}^{p}$ )

In the following, we will focus on *r* ^β^. We see in Figure 5 (left panel) that when the founders consist of individuals from two differentiated populations, the relation between estimates of kinship from markers and predicted values from pedigrees is noisy (*r* ^w^ and *r* ^u^ show a similar pattern; data not shown). In particular, for pedigree kinships of 0, 0.125 and 0.25, we see a large range of marker‐based estimates, larger than when the founders come from a single population (Figure 2). This is because the pedigree predictions *r* ^p^ assume all founders to be equally unrelated, whereas in reality, they are not: Pairs of founders coming from the same population are more related than pairs of founders coming from different populations.

If genetic information is available for the founders, we can account for the heterogeneity of kinship among them by using their marker‐based estimates of kinship as a seed to the algorithm calculating pedigree kinship. This is represented in Figure 5 (right panel). By seeding the pedigree kinship matrix with marker‐based estimates of kinship *r* ^β^ for the founders, the scatter around the pedigree value is much reduced.

**Figure 6:**
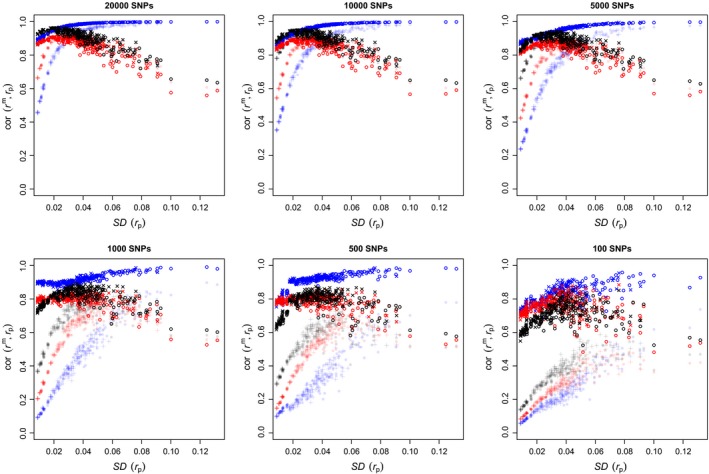
A reproduction of Figure 4 reproduced (in reduced opacity) with the addition of results obtained using markers to estimate founder kinship. Blue: *r* ^β^; red: *r* ^w^; and black: *r* ^u^. Open circles: monogamous pedigrees, where founders’ pedigree kinship was obtained from markers rather than assumed to be 0; *x*: random‐mating pedigrees, where founders’ pedigree kinship was obtained from markers rather than assumed to be 0. Filled circles: monogamous pedigrees. +: random‐mating pedigrees [Colour figure can be viewed at http://wileyonlinelibrary.com]

We can use the same principle when all founders come from one population. The scatter seen in Figure 2 is due to the founders being considered as identically unrelated. If we use the genotypes of the founders to estimate their kinship rather than assuming it to be 0, we obtained Figure 6. The correlation between *r* ^β^ and *r* ^p^ is much increased in all situations, and for the smaller number of typed SNPs (bottom row), *r* ^β^ is the most correlated with *r* ^p^.

### 3.4 Real data applications

#### 3.4.1 Great tit data set

The great tit pedigree is shallow, covering at most three generations, pedigree‐based predictions of kinship have few categories, and some of these have very few observations:

**Table:** 

*r* ^p^	0	0.015625	0.03125	0.0625	0.125	0.25
Number	3,114,008	7	111	289	781	1,060

**Figure 7:**
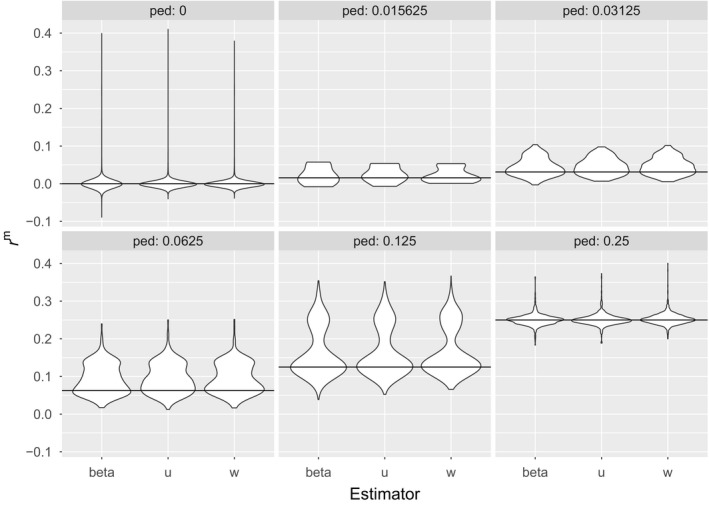
Great tit data. Violin plots of the marker‐based estimates of kinship from *r* ^β^, *r* ^u^ and *r* ^w^ for each level of pedigree kinship *r* ^p^ = 0, (1/2)^*k*^, *k* ∊ [6:2]. Solid lines give the pedigree kinship

Figure 7 displays violin plots of marker‐based estimates of kinship as a function of the pedigree‐based predictions. The three marker‐based kinship estimators show very similar behaviour in this shallow pedigree. The modes of their distributions are aligned with the corresponding pedigree values (horizontal solid lines). Noteworthy is the fairly high proportion of predicted half‐sibs from the pedigree (*r* ^p^ = 0.125) who are identified as full‐sibs with marker‐based estimates of kinship (*r* ^m^ = 0.25, middle panel of the bottom row). A similar pattern is seen for first cousins and half‐sibs. The three marker‐based estimators of kinship for the unrelated individuals show a unimodal distribution with all modes at *r* = 0, but long tails extending to 0.4. This is probably due to founders being related. For instance, individuals 17 and 557, both males and founders, have a pedigree‐based kinship assumed to be 0, but their estimated $r_{17,557}^{\beta }=0.32$. Descendants of these individuals will have their pedigree‐based kinships underestimated.

#### 3.4.2 Soay sheep data set

**Figure 8:**
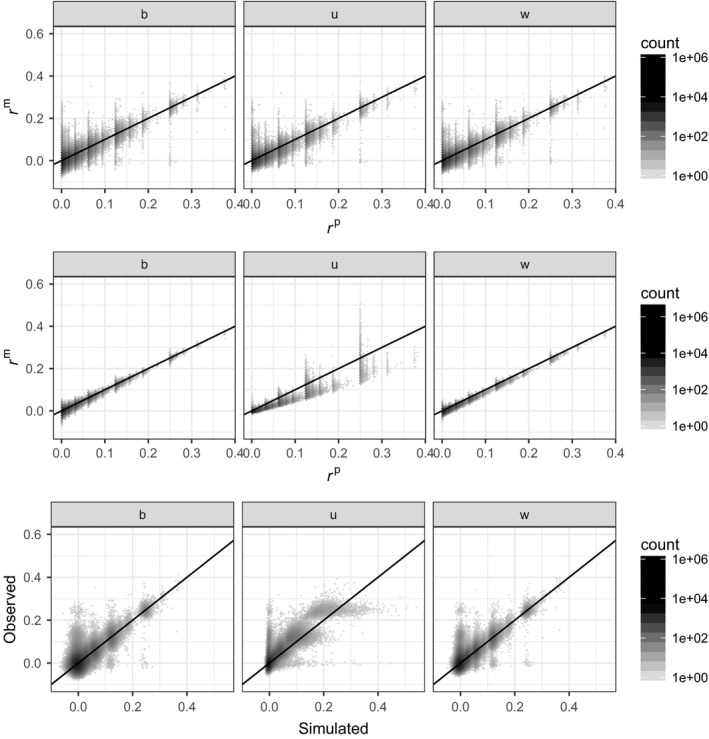
Soay sheep data. Density plots (darker means the more points in the hexagon, and density is on log 10 scale). Top row: marker‐based kinship estimates against pedigree‐based predictions. Middle row: the same as top row, but for simulated genetic data, assuming all founders or unknown parents are unrelated. Bottom row: real marker estimates vs. simulated marker estimates. Left column (b): *r* ^β^; middle column (u): *r* ^u^; and right column (w): *r* ^w^

Next, we look at the sheep data set. Figure 8 presents the results. The top row shows the relation between pedigree‐based predictions of kinship and marker‐based estimates, *r* ^β^, *r* ^u^ and *r* ^w^ from left to right. The correlations are 0.65, 0.73 and 0.71, respectively. While in all three panels we see a tendency for marker‐based estimates to increase with the pedigree‐based predictions, there is much scatter. In particular, for all three marker‐based estimators, some pairs of individuals assumed to be unrelated with pedigree‐based predictions have fairly high marker‐based estimates, and some individuals with pedigree‐based predictions of 0.25 (full‐sibs or parent–offspring) have marker‐based estimates around 0.

The second row of Figure 8 shows the relation between pedigree‐based predictions and marker‐based estimates from genotypes simulated along the pedigree. The relation is much tighter, particularly for *r* ^β^ and *r* ^w^, while *r* ^u^ shows similar scatter to previously, particularly for pedigree kinship class 0.25.

The last row of Figure 8 compares the marker‐based estimates of kinship based on simulations and observed data. For *r* ^β^ and *r* ^w^, most of the points fall close to the one‐to‐one line, and points outside this envelope are easy to identify (for instance, the points for which $r_{\mathrm{dat}}^{\beta }\approx 0.25$ and $r_{\mathrm{sim}}^{\beta }\approx 0$, or those for which $r_{\mathrm{dat}}^{\beta }<0.1$ while $r_{\mathrm{sim}}^{\beta }\approx 0$, bottom left), providing the opportunity to correct the pedigree. It would be much more difficult to use *r* ^u^ for such a correction.

#### 3.4.3 Pig data set

**Figure 9:**
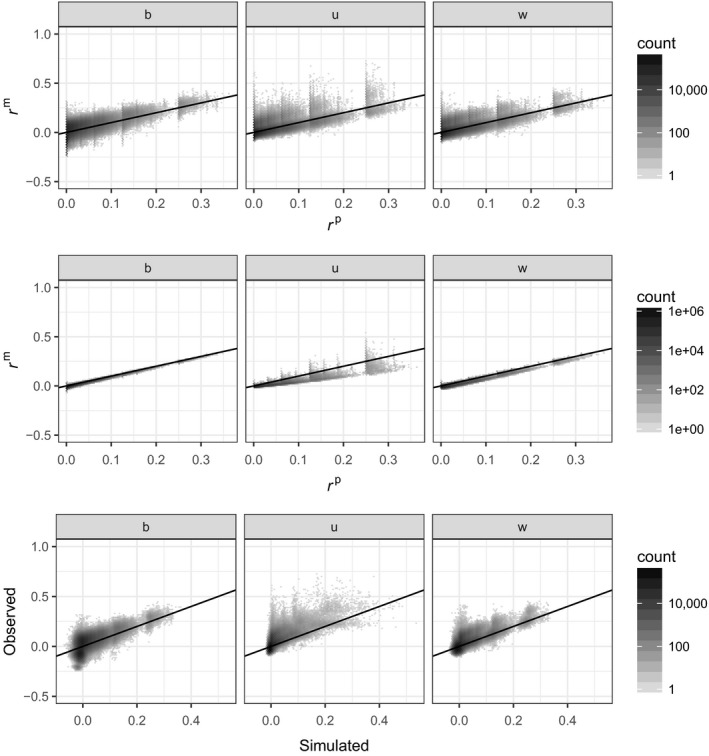
Pig data. Density plots (darker means more points in the hexagon, and density is on log 10 scale) First row: marker‐based estimates of kinship from real data vs. pedigree kinship. b: pedigree kinship vs. *r* ^β^. u: pedigree kinship vs. *r* ^u^. w: pedigree kinship vs. *r* ^w^. Second row: marker‐based estimates of kinship from simulated genetic data vs. pedigree‐based kinship. b: pedigree kinship vs. *r* ^β^. u: pedigree kinship vs. *r* ^u^. w: pedigree kinship vs. *r* ^w^. Third row: real vs. simulated marker‐based kinship. β: *r* ^β^. u: *r* ^u^. w: *r* ^w^

This data set is the most complete: All individuals (bar founders) have both parents identified. Close to 55% of the 6,473 individuals in the pedigree have been genotyped. The first row of Figure 9 shows the relation between pedigree‐ and marker‐based kinship for the three marker‐based estimators. The relation is not as tight as that seen in Figure 2, and the correlation between marker‐ and pedigree‐based kinships is 0.55, 0.55 and 0.56 for *r* ^β^, *r* ^u^ and *r* ^w^, respectively, for a standard deviation of pedigree‐based kinship estimates of 0.02. There is therefore little separating the three marker‐based estimators of kinship in terms of correlation, and *r* ^β^ shows the least scatter and bias of the three estimators (compare the top left panel (b) of Figure 9 with the top middle (u) and right (w) panels).

The marker‐based estimates of kinship (particularly *r* ^β^) from simulated genetic data (middle row of Figure 9) match the pedigree‐based kinships extremely well. The correlation between marker‐based and pedigree‐based predictions is 0.96, 0.85 and 0.87 for *r* ^β^, *r* ^u^ and *r* ^w^, respectively (the results are almost identical if using only 42*k* SNPs; filtering on minor allele frequency (MAF) larger than 0.01 reduces the correlation for *r* ^β^ and *r* ^w^ and marginally increases it (0.85 to 0.87) for *r* ^u^). The last row of Figure 9 shows the relation between simulated marker‐based estimates of kinship and observed marker‐based estimates of kinship. The key point to take from this bottom row is best seen from the leftmost panel comparing the two *r* ^β^: Among the simulated marker‐based estimates of kinship close to 0, we observed two high‐density spots of estimates from real data (the two dark spots), an indication that the founders may not come from a homogeneous stock.

Seeding the pedigree‐based estimator of kinship with the marker‐based estimation of kinship for the 81 genotyped founders does not significantly improve the relation between marker‐ and pedigree‐based values (data not shown).

## 4 DISCUSSION

For three data sets where both pedigree and genetic data are available, the match between pedigree‐based predictions and marker‐based estimates of kinship is poor. Using simulated pedigrees and genetic data, we identify two likely causes for this mismatch: errors in the assignment of parentage when constructing the pedigree and heterogeneity in the origin of the founders. We show that the new estimator *r* ^β^ closely tracks *r* ^p^ over the whole range of kinship values (despite being an estimator of β, not *r* ^p^) and performs better than *r* ^u^ and *r* ^w^ for small pedigrees and pedigrees with many related individuals. *r* ^u^ is a poor estimator of *r* ^p^ for pairs of individuals with high kinship. We confirm that with a sufficient number of markers, marker‐based estimation of kinship better reflects individual relationship than pedigree‐based prediction.

Heterogeneity in the origin of founders seems quite clear in the pig data set (see bottom row of Figure 9) and the Soay sheep data set (bottom row of Figure 8). This has been discussed for the sheep by Feulner et al. (2013) who suggest that the 107 Soay sheep introduced on the island of Hirta in 1932 were the result of admixture between Soay and Dunface sheep. The founders of the pedigree analysed here are more recent (around the 1980–1990s), and it is unclear whether more recent admixtures or introductions took place after 1932. Heterogeneity of genetic origin among founders is likely to be common in populations (re)established for conservation, and it seems that much is to be gained by using genetic markers rather than pedigrees in these situations.

A third source of discrepancy between marker‐ and pedigree‐based values of kinship is the size of the genome: With small genome size, genetic relatedness will differ from pedigree‐based expectations (Hill & Weir, 2011). This holds true even with a very large number of SNPs since these SNPs are inherited as blocks (Wang, 2016). We have assumed unlinked markers in our simulations, effectively assuming infinitely large genomes. Pigs and sheep have approximately 2.7 Gb genomes (Groenen et al., 2012; Jiang et al., 2014), and the tit genome is around 1 Gb (Cai et al., 2013). Accordingly, pig and sheep genomes are ≈ 20 morgans long, and the tit genome is smaller. Figure 1 of Wang (2016) shows that the correlation between pedigree‐based prediction and true kinship is around 0.9 for genome that is ≈ 20 morgans long. Thus, part of the scatter seen in Figure 1 may result from finite genomes. To verify this, We ran additional simulations with a finite genome of 20 morgans instead of an infinite‐sized genome, the results are shown in Supporting Information Figure S5 and are essentially the same as Figure 3.

Another potential source of discrepancy between marker and pedigree estimates of kinship is the type of genetic data used. The three observed genetic data sets were obtained from DNA array data, which typically focus on common variants and filter out the rarest. We found with the pig data set that filtering on MAF slightly reduces the correlation for *r* ^β^ and *r* ^w^ and only marginally increases it for *r* ^u^. This is in agreement with the findings in table 7 of Weir and Goudet (2017), where increasing levels of MAF filtering (from 0.01 to 0.1) increased the downward bias of *r* ^β^. We thus recommend the use of the full range of the allele frequency spectrum whenever possible when estimating *r* ^β^.

### 4.1 Properties of marker estimates

Figure 3 shows that the correlation between *r* ^β^ and pedigree kinship increases as the variance in pedigree kinship increases. This makes intuitive sense. If all individuals are unrelated, there is no variation in kinship and nothing to explain. As the proportion of related individuals increases, genetic similarity between individuals becomes a good proxy for kinship, and this tendency should increase with the proportion of related individuals. Seeing the correlations decrease after the ≈ 0.02 threshold of standard deviation in pedigree kinship for *r* ^u^ and *r* ^w^ is initially puzzling. However, these two estimators were derived assuming the data come from a random‐mating population (Ritland, 1996). If this is not the case, then the expectations of $r_{jj^{\prime }}^{u}$ and $r_{jj^{\prime }}^{w}$ are not the kinship of individuals *j* and *j*′, but a complex function of their kinship and their average kinship with all other individuals in the population, as shown in Equation (3) and demonstrated in Weir and Goudet (2017).

Substantial improvements in the estimation of *r* ^p^ (rather than γ) by *r* ^u^ and *r* ^w^ could be obtained by using the founders’ allele frequencies (if these were available) instead of the sample allele frequencies (VanRaden et al., 2011). In many ecological situations, however, it is not possible to extract the frequencies from the founders generation, either because the founders are not known and no pedigree is available or because no genetic information is available from the founders (for instance, genetic data were available for only 81 of the founders in the pig data set).

The third method to estimate kinship described in VanRaden (2008) contains MM′ as the central quantity and, like *r* ^β^, does not require estimation of allele frequencies. In this method, the intercept of the regression of MM′ on pedigree kinship is subtracted, thus ensuring that the mean value of marker‐based kinship for individuals whose pedigree kinship is 0 is also 0. This difference is then divided by the slope of the regression of MM′ on pedigree kinship, constraining the upper bound of the third estimate to 1. *r* ^β^ differs from this third estimate in that the mean of all MM′ values (excluding the diagonal elements), *M*_S_, is subtracted from each entry, ensuring that the overall mean kinship is 0. We then divide this difference by 1 − *M*_S_, constraining the upper bound of *r* ^β^ to 1. In order to obtain the third estimate of VanRaden (2008), one needs both pedigree and markers, while calculating *r* ^β^ requires neither a pedigree nor allele frequencies.

In the animal breeding literature, including both genomic estimates of kinship and pedigree values for genomic prediction is a very active question. The key difference with the ecological and evolutionary biology literature is the reliability of the pedigrees (in terms of accuracy, completeness and depth). In ecological and evolutionary biology studies, pedigrees are never as complete and accurate as in animal breeding, and inclusion of pedigree information is likely to add more noise than information.

### 4.2 Which marker‐based estimate of kinship should be used?

There is not a general answer to this question. Below we list context‐dependent recommendations:

- Where founder populations are small (e.g., reintroductions and threatened populations), the recommended marker‐based estimate of kinship is *r* ^β^.
- In sample populations with high kinship (such as those used to investigate cooperation between kin), *r* ^β^ is the recommended estimator of kinship, as it performs well across the whole range of kinship values. At the level of first cousins and above, *r* ^u^ performs poorly.
- For estimation of heritability and variance components in genomewide association studies, the situation is more complicated and will depend on population parameters: If the population is large enough that the individuals analysed are unlikely to be related above the level of first cousins, *r* ^u^ should be used to estimate the genetic relationship matrix, since it estimates the kinship of unrelated individuals most accurately. It might be necessary in this case to filter the SNPs for MAF larger than 1%, as low‐frequency SNPs tends to generate a long tail of high kinship values for unrelated individuals (see Supporting Information Figure S2, top left subpanels in each panel). If the population is not so large or higher levels of kinship are suspected, the data set could be first filtered to remove these individuals (they likely share more than their genotypes, i.e., some maternal and environmental effect), or one could use *r* ^β^ or *r* ^w^ to estimate the genetic relationship matrix. We also note that when kinship is estimated to calculate the heritability of a trait for a specific genomic region, Speed, Hemani, Johnson, and Balding (2012) showed that estimation is improved by accounting for and borrowing from SNPs near the focal region. In this context, such methods might be more relevant than the estimates presented here.

### 4.3 Take‐home messages

Finally, our results point to the following:

- As identity by descent is not an absolute state, but is relative to a reference population for which there is generally little information, we can estimate the kinship of a pair of individuals only relative to some other quantity. For *r* ^β^, we use the average kinship of all pairs of individuals in a study as the reference value.
- With 10*k* SNPs or more, marker‐based estimates of kinship perform very well.
- When using pedigrees, completeness is paramount. Even in complete pedigrees, pedigree kinship expectations may differ dramatically from true genetic kinship if founders come from different populations.
- *r* ^u^ is accurate if very few individuals are related, but estimates kinship for closely related individuals (first cousins and above) poorly. *r* ^β^ is accurate over the whole range of possible kinship values.
- The recommended marker‐based estimate of kinship to use depends on the amount of variation in kinship in the population.
- MAF filtering is not a good idea; it diminishes the correlation with pedigree kinship in most cases.
- The most suitable marker‐based estimator also depends on why kinship is being estimated. If the purpose is estimation of heritability, and the proportion of related individuals is small, *r* ^u^ is a good choice. For choosing breeders in conservation genetics or for comparing levels of relatedness among pairs of individuals in social species, *r* ^β^ is the estimator of choice.

## AUTHOR CONTRIBUTIONS

J.G. and B.S.W. conceived the study. T.K. carried out preliminary analyses as part of his master; J.G. wrote the paper, with contributions from all authors.

## DATA AVAILABILITY

Data available from the Dryad Digital Repository: https://doi.org/10.5061/dryad.ds8fk04.
