# Supplementary material for: How to estimate kinship
Source: Mol Ecol. 2018 Sep 7;27(20):4121–35. doi: 10.1111/mec.14833 (PMC6220858; doi:10.1111/mec.14833)
Supplement: Supplementary file 1 [file MEC-27-4121-s001.pdf]

SUPPLEMENTARY NOTES TO "HOW TO ESTIMATE KINSHIP"

| Founder's genotypes

Hudson's `ms` was used to generate the genotypes of the founders only. These were obtained using the command `ms 2000 1000 -t 10`, i.e. 1000 independent replicates of a set of fully linked SNPs, the number of fully linked SNPs varying between 51 and 159 depending on the replicate (mean=88.1). While subsets of SNPs are linked in the founders genomes, they are independent for all the following generations in the pedigree, as explained in the text.

| Relation between the number of individuals in a pedigree and $SD(r_p)$

Figure S1 illustrates the relation between the number of individuals in a pedigree and the standard deviation in pedigree kinship $SD(r_p)$. The fewer individuals in a pedigree, the larger $SD(r_p)$, and pedigrees generated under monogamous mating have larger variation in kinship than those generated under random-mating.

| Violin plots for simulated pedigrees

Figure S2 illustrates the behavior of the three estimators for given categories of pedigree kinship. We extracted from the simulated pedigrees with 250 and $1,000$ founders all the pairs of pedigree kinship values $r^p = 0, (1/2)^k, k \in [6, 2]$. These correspond to unrelated individuals, third degree cousins, up to full-sibs or parent-offspring. The top panel is for pedigrees with 250 founders, the bottom panel for $1,000$ founders, monogamous matings on the left and random mating on the right. Each subpanel displays the violin plots of the three estimators r^β, r^u and r^w for each r^p value. For unrelated pairs (top-left subpanel in each panel), r^u is the least variable (widest violin) but shows a tail of large values. For full-sibs or parent-offspring (lower right subpanel), r^β is the least variable and biased, followed closely by r^w. r^u in this situation is downwardly biased and shows a very large variance in all four panels. It is still poorly behaved for first cousin pairs.

| Handling missing data

While it might be practical for small data sets to discard missing genotypes in the estimation of r^β, for large datasets this imposes a very large computing cost. Each locus will have a different individual count, which will prevent the use of matrix operations, or at least make them much more complicated to implement.

An alternative solution is to impute the missing genotypes. Several solutions exist when a genetic map is available Marchini and Howie (2010), but here we will focus on the situation where such a map does not exist.

A common and simple solution is to assign to missing values the mean of the observations, but this introduces biases and reduces the variance, as well as the covariance among observations (e.g. Horton and Kleinman (2007)).

To illustrate this, we use one of the pedigrees discussed earlier (see Figure S3). It consists of $1,454$ individuals genotyped at $28,000$ SNPs. The first 250 individuals are founders, coming from two populations with $F_{ST} = 0.112$. We first compare the estimated r^β for the data set with no missing data to that with the same data set with 1, 5, 10 and 20% missing data, generated completely at random (top left panel of Figure S3). In each case, the mean allelic dosage across all individuals at the considered locus replaces the missing data.

The more missing data, the more biased the kinship estimates, and the further from the mean, the larger is the bias. The variance is also reduced: a well known behavior when missing values are replaced by the mean of the observations (Horton and Kleinman, 2007). The bias is proportional to the proportion of missing data.

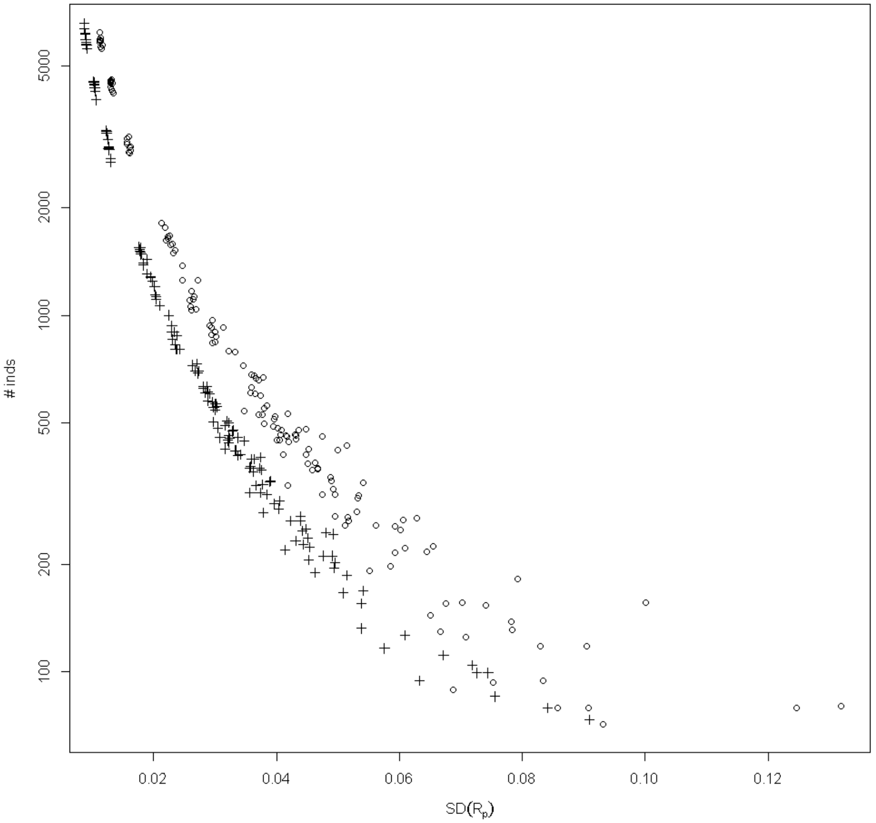

FIGURE S1 The empirical relation between the number of individuals in a pedigree and $SD(r_p)$, the standard deviation in pedigree kinship. Circles: monogamous mating; + random mating. Each point corresponds to one of the simulated pedigrees

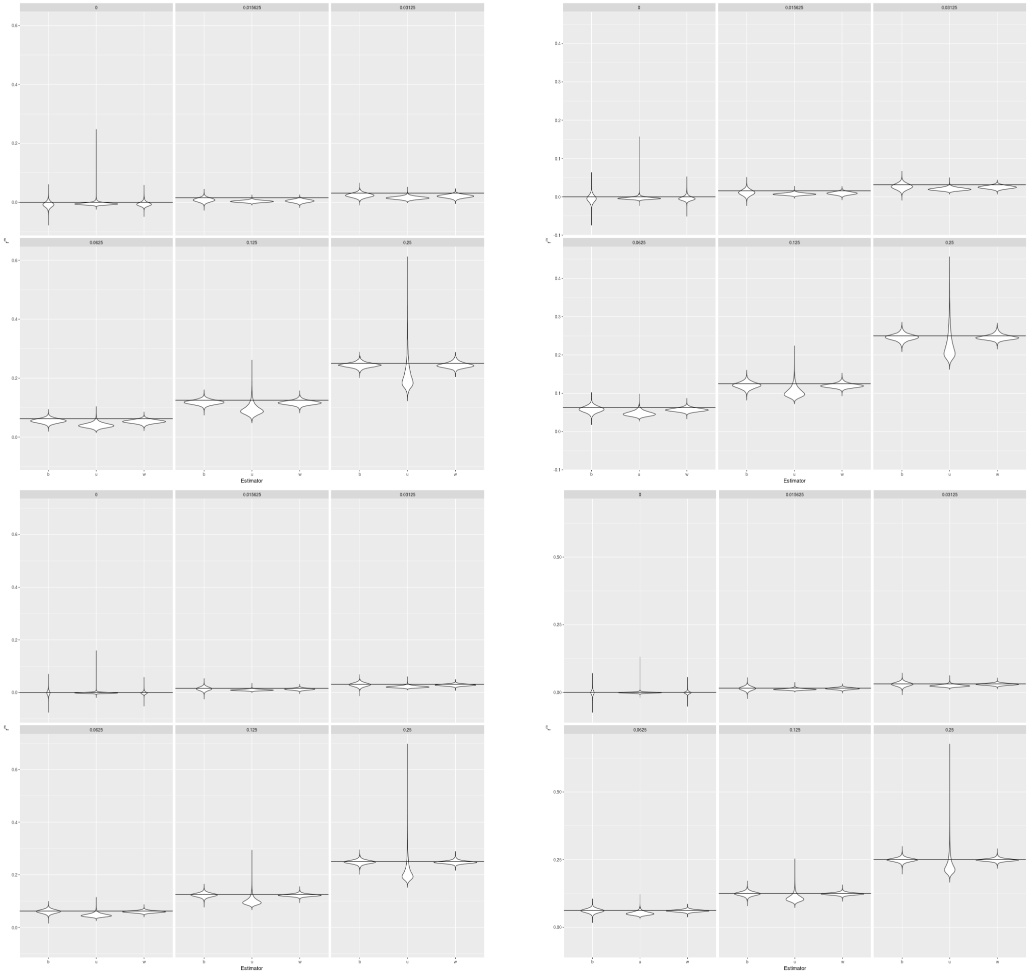

FIGURE S2 Violin plots of specific pedigree kinship classes for pedigrees with 250 (top) and 1000 (bottom) founders. Left: monogamous pedigrees; right: random mating pedigrees

We next divide each pairwise kinship by the product of the proportion of non-missing data for each individual:

$$r_{i,j}^{\beta_c} = \frac{r_{i,j}^{\beta}}{(1 - m_i)(1 - m_j)}$$

where m_i and m_j are the proportions of missing data for individuals i and j respectively. We replace all missing values by the mean frequencies. r^β contains a cross-product. We are thus bringing each observation closer to the mean by a proportion $(1 - m_i)(1 - m_j)$. By dividing by this last quantity, we restore the initial value. In this sense, what we are doing is not really imputing, but using a efficient way to calculate our estimator with missing values. The results of this correction are shown on the top right panel of Figure S3. VanRaden (2008) suggests a similar method to account for missing dat.

We also found that the estimates of the individual inbreeding coefficients F_i, when missing data are imputed as the mean of the locus, are strongly downwardly biased, and show reduced variance (the slope of the regression of F_i with missing values on f_i without missing values is less than 1) (Bottom left panel of Figure S3). The downward bias is exactly the proportion of missing data, and the reduction of variance is also a function of the proportion of the missing data. Hence, an *ad hoc*, corrected estimate ($F_i = r_{ii}^{\beta} * 2 - 1$. The -1 is in fact $-(1 - m_i)$ since we have a proportion m_i missing and the correlation is dampened by a factor $(1 - m_i)$), when there is missing data for the inbreeding coefficient is:

$$\hat{F}_i^c = \frac{\hat{F}_i + m_i}{1 - m_i}$$

The bottom right panel of Figure S3 illustrates the effect of the correction on the inbreeding coefficient.

| Pig data set violin plot

Figure S4 shows violin plots of the marker-based estimates of kinship for a subset of the pedigree-based values. r^β estimates have distributions that differ from r^u and r^w. It is particularly striking for the full-sibs/ parent-offspring category (bottom right panel), where r^β seems unimodal whereas the two other marker based estimates are bimodal, the second mode being larger than the expected value of 0.25.

| Correlation between marker and actual kinship for finite size genome

We ran additional simulations with the same pedigrees as in the main text, but with a finite genome of 20 Morgans. Rather than using pedigree predicted kinship, we quantified the actual kinship. To this end, each founder was assigned two unique identifiers at each of $20k$ loci. For each individual in the pedigrees, gametes were drawn randomly from each parent, drawing crossing-over numbers from a Poisson distribution with mean 20 and crossing-over positions from a uniform distribution. Actual kinship r^g was then estimated for each of the $20k$ loci as a quarter of the number of matches between the four pairs of alleles, and then averaged over loci.

Genotypes for the gametes of the founders at $20k$ SNPs were generated using `ms`, assuming a map of 20 morgans. The unique identifiers allocated to each founder at each locus were then mapped to the gamete's genotype generated with `ms`, and this mapping was used to obtain the genotypes of all individuals in the pedigree.

Last we calculated r^β, r^w and r^b as in the main text, and computed their correlation with actual kinship r^g. The results are presented in figure S5 and are essentially the same as those shown in figure 3.

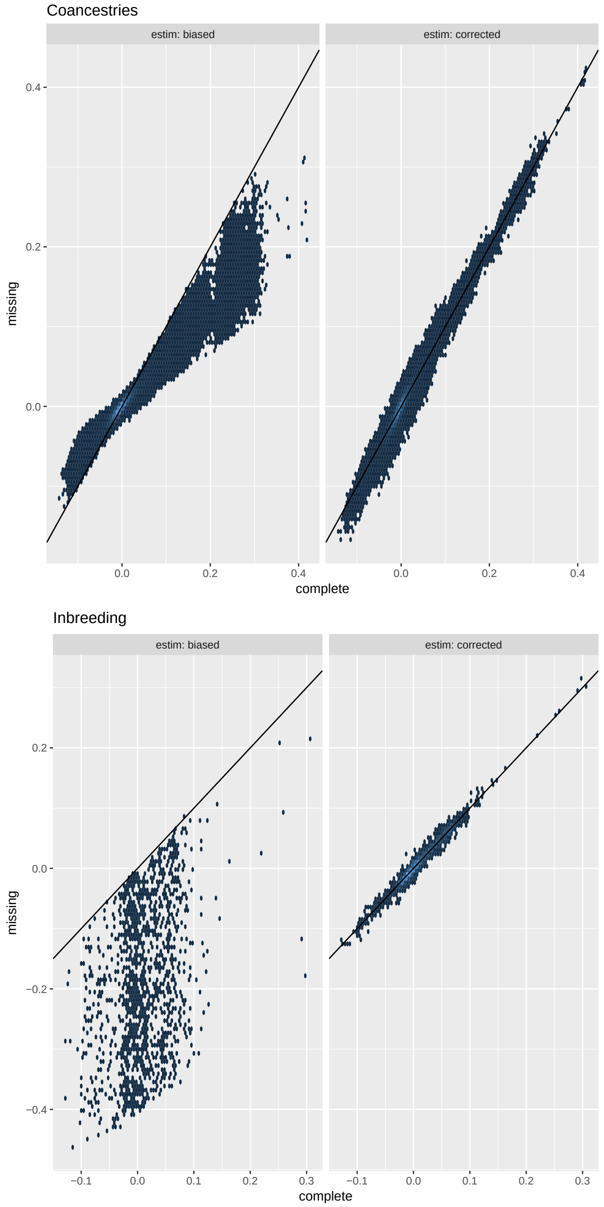

FIGURE S3 Top: effect of the correction for the kinship coefficient. r^{β} with missing data as a function of r^{β} without missing data. Left panel: uncorrected estimate of **[kinship]** . Right panel: corrected estimate of kinship. Bottom: effect of the correction for the inbreeding coefficient. F^{β} with missing data as a function of F^{β} without missing data. Left panel: uncorrected estimate of inbreeding. Right panel: corrected estimate of inbreeding

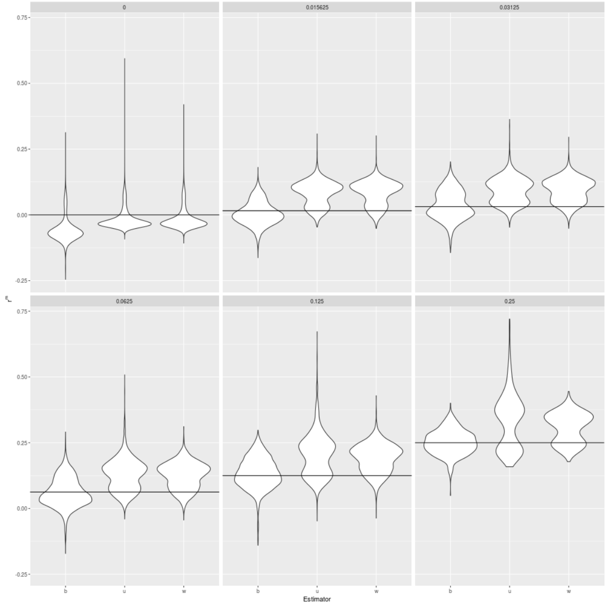

FIGURE S4 Pig data set: violin plots of the three marker-based estimates of kinship for a subset ($r^p = (0, (1/2)^k), k \in [6, 2]$) of the pedigree based kinship **[values]**

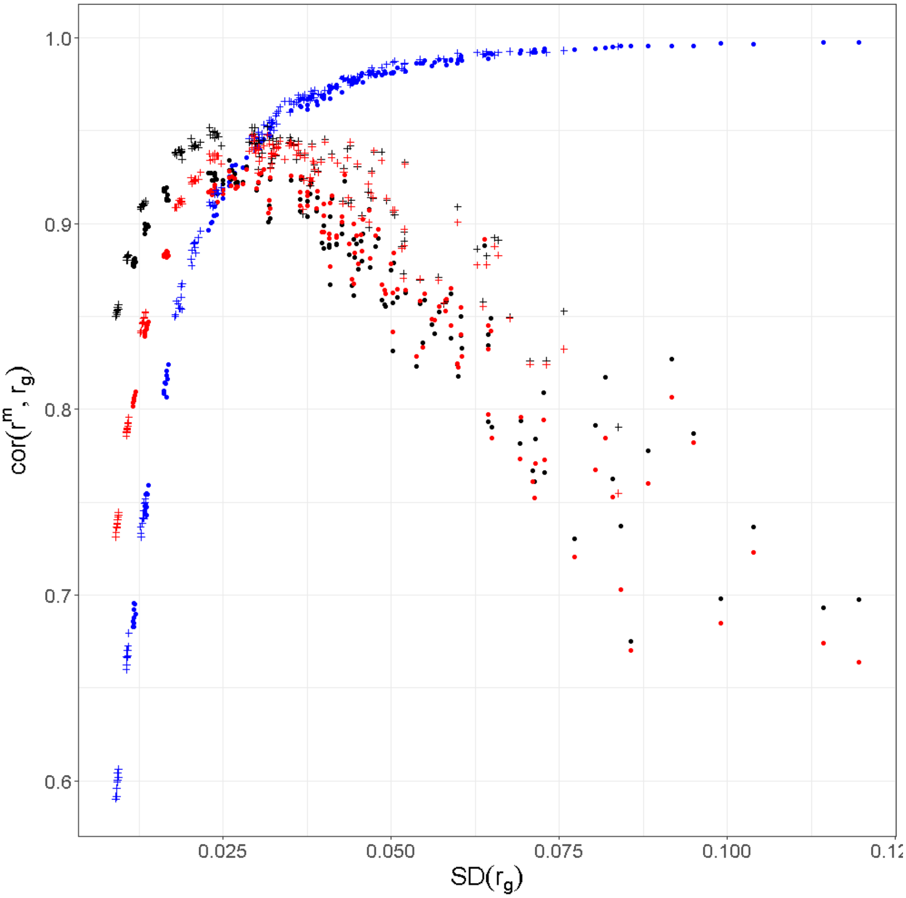

FIGURE S5 Correlation between marker-based kinship with 20k SNPs and actual values r^g, against the standard deviation of actual kinship $SD(r^g)$. Each point corresponds to one of the 300 simulated pedigrees. Blue: r^β; red: r^w; black: r^u. Filled circles: monogamous pedigrees. +: random-mating pedigrees.
